# Continuously assessed right ventricular end-diastolic volume as a marker of cardiac preload and fluid responsiveness in mechanically ventilated cardiac surgical patients

**DOI:** 10.1186/cc3503

**Published:** 2005-04-01

**Authors:** Christoph Wiesenack, Christoph Fiegl, Andreas Keyser, Sven Laule, Christopher Prasser, Cornelius Keyl

**Affiliations:** 1 Consultant, Department of Anesthesiology, University Hospital Regensburg, Regensburg, Germany; 2 Resident, Department of Anesthesiology, University Hospital Regensburg, Regensburg, Germany; 3 Staff Surgeon, Department of Cardiothoracic and Vascular Surgery, University Hospital Regensburg, Regensburg, Germany; 4 Staff Anesthesiologist, Department of Anesthesiology, Heart-Center Bad Krozingen, Bad Krozingen, Germany; 5 Consultant, Department of Anesthesiology, Heart-Center Bad Krozingen, Bad Krozingen, Germany

## Abstract

**Introduction:**

Assessing cardiac preload and fluid responsiveness accurately is important when attempting to avoid unnecessary volume replacement in the critically ill patient, which is associated with increased morbidity and mortality. The present clinical trial was designed to compare the reliability of continuous right ventricular end-diastolic volume (CEDV) index assessment based on rapid response thermistor technique, cardiac filling pressures (central venous pressure [CVP] and pulmonary capillary wedge pressure [PCWP]), and transesophageal echocardiographically derived evaluation of left ventricular end-diastolic area (LVEDA) index in predicting the hemodynamic response to volume replacement.

**Methods:**

We studied 21 patients undergoing elective coronary artery bypass grafting. After induction of anesthesia, hemodynamic parameters were measured simultaneously before (T1) and 12 min after volume replacement (T2) by infusion of 6% hydroxyethyl starch 200/0.5 (7 ml/kg) at a rate of 1 ml/kg per min.

**Results:**

The volume-induced increase in thermodilution-derived stroke volume index (SVI_TD_) was 10% or greater in 19 patients and under 10% in two. There was a significant correlation between changes in CEDV index and changes in SVI_TD _(r^2 ^= 0.55; *P *< 0.01), but there were no significant correlations between changes in CVP, PCWP and LVEDA index, and changes in SVI_TD_. The only variable apparently indicating fluid responsiveness was LVEDA index, the baseline value of which was weakly correlated with percentage change in SVI_TD _(r^2 ^= 0.38; *P *< 0.01).

**Conclusion:**

An increased cardiac preload is more reliably reflected by CEDV index than by CVP, PCWP, or LVEDA index in this setting of preoperative cardiac surgery, but CEDV index did not reflect fluid responsiveness. The response of SVI_TD _following fluid administration was better predicted by LVEDA index than by CEDV index, CVP, or PCWP.

## Introduction

Accurate evaluation of cardiac performance and preload status, and assessment of fluid responsiveness are important goals in the treatment of critically ill patients. Despite the current controversy surrounding the usefulness of and risks associated with the pulmonary artery catheter (PAC) [1,2], the PAC remains more frequently used for monitoring and is preferred over transesophageal echocardiography (TEE) by cardiovascular anaesthesiologists [3]. However, it has been demonstrated that PAC-derived filling pressures are of little help when making decisions regarding adequate volume therapy. Nevertheless, the majority of intensive care unit (ICU) physicians use filling pressures in their decision making regarding volume replacement to improve hemodynamics. This accentuates the need for reliable indicators of fluid responsiveness so that needless or even deleterious volume replacement associated with increased morbidity and mortality may be avoided in critically ill patients [4]. Several markers of ventricular preload, specifically intrathoracic blood volume [5,6], TEE-derived assessment of left ventricular end-diastolic area (LVEDA) [7,8], and cyclic fluctuation in arterial pressure wave that occurs in mechanically ventilated patients [7,9-14], have been tested as predictors of fluid responsiveness, some with excellent results. However, apart from pulse contour analysis, which has never been found in positive-pressure ventilation to reflect actual stroke volume variation [15,16], none of the techniques for assessing preload can be used continuously or routinely in most patients.

Several studies have emphasized the good correlation between estimates of right ventricular end-diastolic volume (RVEDV) by thermodilution-derived right ventricular ejection fraction (RVEF) and surrogates of stroke volume [17-20]. However, the thermodilution technique for assessing RVEDV is still intermittent, and the value of RVEDV as a marker of fluid responsiveness in critically ill patients is controversial [20-22].

A recently available Swan–Ganz catheter with a rapid response thermistor permits nearly continuous assessment of cardiac output (CO), RVEF and RVEDV, which should be more applicable in the ICU. The measurement variability associated with the intermittent bolus technique is eliminated by this catheter, and continuously assessed RVEDV (CEDV) should be more accurate than RVEDV based on intermittent thermodilution; therefore, CEDV may be a valuable marker of cardiac preload and a predictor of fluid responsiveness.

The purpose of the present study was to compare the accuracy of CEDV derived using a new right-heart ejection fraction catheter and commonly used preload parameters (central venous pressure [CVP], pulmonary capillary wedge pressure [PCWP], and transesophageal echocardiography [TEE]-derived assessment of LVEDA) in predicting the response of stroke volume to volume replacement in mechanically ventilated cardiac surgical patients.

## Materials and methods

After obtaining approval from the local ethics committee and written informed consent from all participants, we studied 21 patients (17 male; aged 53–78 years, mean 65.7 years) undergoing elective coronary artery bypass grafting. Patients with valvular heart disease, intracardiac shunts, regional myocardial asynchrony, peripheral vascular disease, preoperative dysrhythmias, and an ejection fraction under 30 % were excluded from the study. Dynamic variables, such as pulse pressure variation, were not measured to assess fluid responsiveness in our investigation.

All patients received an arterial catheter for continuous monitoring of arterial blood pressure (Siemens monitor SC 9000; Siemens AG, Erlangen, Germany). Anesthesia was induced with fentanyl (5 μg/kg) followed by etomidate until loss of consciousness and pancuronium (100 μg/kg), and maintained using 1.5% sevoflurane end-expiratory, supplemented with bolus doses of fentanyl (up to 20 μg/kg) and pancuronium (50 μg/kg) for neuromuscular blockade. Mechanical ventilation (without positive end-expiratory pressure) at a constant tidal volume of 7 ml/kg to an end-tidal partial carbon dioxide tension of 30–35 mmHg was maintained at a inspired fractional oxygen of 0.5 throughout the study.

After induction of anesthesia, a 7.5 Fr right-heart ejection fraction catheter (CCOmboV 774HF75; Edwards Lifesciences, Irvine, CA, USA) was inserted via an 8.5 Fr introducer into the right internal jugular vein and connected to a Vigilance Monitor system (Edwards Lifesciences) for continuous assessment of CO (CCO), CEDV and of RVEF, and for determination of CO using the intermittent thermodilution technique (CO_TD_).

The methodology of CCO measurement, based on the pulsed warm thermodilution technique, was described previously [23] and involves the release of small pulses of heat from a thermal coil mounted on the PAC at the level of the right ventricle. To reflect sudden changes in CO, the Vigilance Monitor provides a STAT mode of operation, which has been shown to permit accurate measurement of CCO [24]. The software algorithm for STAT CCO does not contain a moving average filter but depends on some previous data for artifact suppression. Without user calibration, CCO is computed from the area under the thermodilution curve, and every 30–60 s the displayed CCO is updated.

**Figure 1:**
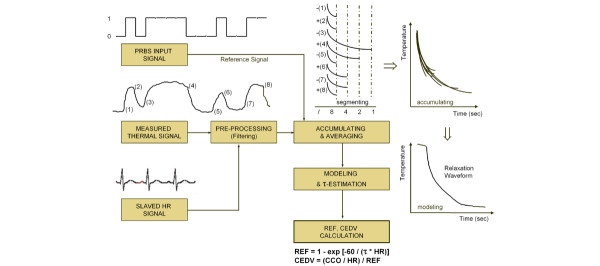
CEDV assessment. Shown is a modified algorithm block diagram for continuous right ventricular end-diastolic volume (CEDV) assessment. CCO = continuous cardiac output; CEDV = continuous right ventricular end-diastolic volume; HR = heart rate; PRBS = Pseudo-Random Binary Sequence; REF = right ventricular ejection fraction; τ = exponential decay time constant. Courtesy of Edwards Lifesciences, Unterschleissheim, Germany.

The new CEDV algorithm uses the slaved electrocardiograph signal and generates a relaxation waveform, which resembles the bolus thermodilution washout decay curve. The waveform is based on the repeating on–off CCO input signal and is generated by accumulating the temperature change for each on and each off segment of the input signal (Fig. 1). Calculation of RVEF is based on estimation of the exponential decay time constant (τ) of this curve and heart rate (HR): RVEF = 1 - exp (-60/ [τ × HR]). CEDV, which is based on CCO, HR and RVEF, is calculated as follows: CEDV = (CCO/HR)/RVEF. It includes the whole range of temperatures of the thermodilution curve (Fig. 1).

CO_TD _measurements were performed by injection of 10 ml iced saline solution via the CVP port and subsequent detection by the thermistor embedded in the PAC. An average of three measurements, all taken within a 10% range randomly distributed over the respiratory cycle, was calculated using the Stewart–Hamilton formula.

The TEE probe (OmniPlane II probe 21369A and SONOS 5500 Phased Array Imaging System; Philips Medical Systems, DA Best, The Netherlands) was positioned to obtain a transgastric midpapillary short-axis view of the left ventricle. This position was maintained over the whole period of data acquisition. Echocardiographic images and electrocardiograms were recorded together, and end-diastole was defined as the greatest left ventricular cross-sectional area immediately after the R-wave peak on the electrocardiogram.

Correspondingly, end-systole was defined as the smallest left ventricular dimension during the last half of the T wave. An independent reviewer, who was blinded to the condition of the trial participants, analyzed TEE images. LVEDA and left ventricular end-systolic area were traced edge to edge, including the papillary muscles. Fractional area change was calculated as (LVEDA – left ventricular end-systolic area)/LVEDA. Three measurements, performed at end-expiration, were analyzed and averaged.

All hemodynamic parameters were measured simultaneously after induction of anesthesia, when CCO had stabilized (T1). A second measurement was performed (T2) 12 min after volume replacement by infusion of 6% hydroxyethyl starch 200/0.5 (7 ml/kg) at a rate of 1 ml/kg per min (mean 579 ml). Measurements were taken in a hemodynamically steady state, in the absence of vasoactive drugs. Patients were classified as responders to volume loading if the increase in thermodilution-derived stroke volume index (SVI_TD_) was 10% or greater, or as nonresponders if the increase in SVI_TD _was under 10%.

### Statistical analysis

For statistical analysis, all volume variables were indexed to body surface area. Statistical analysis was performed using the SPSS 12.0 software (SPSS Inc., Chicago, IL, USA). After assessment of normal distribution using the Lilliefors modification to the Kolmogorov–Smirnov test, the Student's t-test was used to compare variables. Because the thermodilution technique still represents the 'gold standard' for assessment of cardiac index (CI), we conducted linear regression analyses between changes in variables that reflect preload (CEDV index, CVP, PCWP, and LVEDA index) and changes in the preload-dependent variable SVI_TD_, and between baseline (T1) values of variables that reflect preload (CEDV index, CVP, PCWP, and LVEDA index) and the change in SVI_TD _(ΔSVI_TD_; expressed as a percentage). *P *< 0.05 was considered statistically significant.

## Results

Demographic data for the patients included in the present study are summarized in Table 1.

Except for HR, all hemodynamic parameters changed significantly after volume replacement (Table 2). The volume-induced increase in SVI_TD _was 10% or greater (range 21.8–93.4%) in 19 patients (responders) and under 10% in two patients (nonresponders).

**Figure 2:**
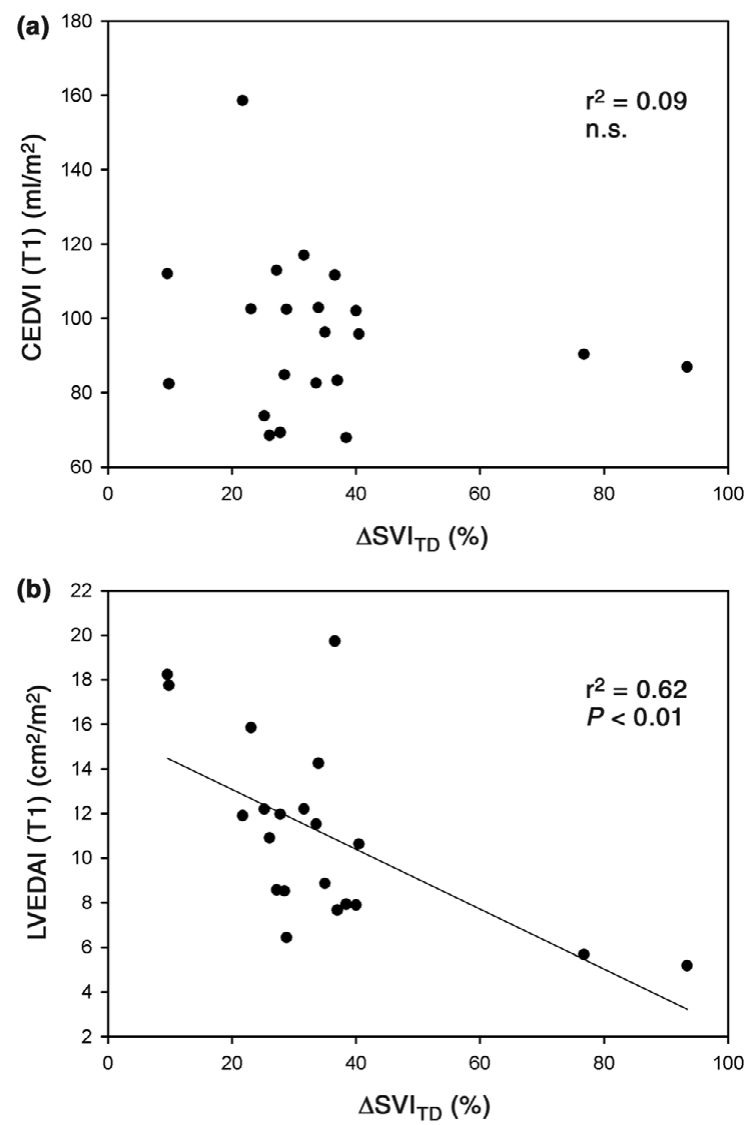
Linear regression analyses. Linear regression analysis between **(a) **changes in thermodilution-derived stroke volume index (ΔSVI_TD_) and baseline values of continuously assessed right ventricular end-diastolic volume index (CEDVI), and between **(b) **ΔSVI_TD _and baseline values of transesophageal echocardiographically derived left ventricular end-diastolic area index (LVEDAI).

Linear regression analysis between changes in CEDV index (ΔCEDV index) and ΔSVI_TD _revealed a significant correlation (r^2 ^= 0.55; *P *< 0.01), but linear regression analysis between changes in CVP, PCWP and LVEDA index, and ΔSVI_TD _did not identify any significant correlations among variables. LVEDA index at baseline and the percentage ΔSVI_TD _were weakly correlated (r^2 ^= 0.38; *P *< 0.01), but linear regression analysis between the remaining variables reflecting preload (CEDV index, CVP, and PCWP) did not reveal any significant relationships (Fig. 2). Variables reflecting systolic function – RVEF and fractional area change – remained constant, without a significant relationship between them.

## Discussion

Over recent years numerous studies have been performed to evaluate the usefulness of thermodilution-derived estimates of RVEDV index in a variety of clinical situations [17-20,25-28]. Several investigators emphasized the good correlation between RVEDV index and CI [17-20], suggesting that a volumetric assessment of cardiac preload may provide a more useful evaluation of ventricular filling than that offered by the assessment of cardiac filling pressures. A previous study found that a RVEDV index greater than 138 ml/m^2 ^was associated with lack of response but that RVEDV index below 90 ml/m^2 ^was associated with a high rate of response to fluid administration [18]. In contrast to these findings, Wagner and Leatherman [22] reported a positive response to volume loading in a number of patients with an RVEDV index above 138 ml/m^2 ^and a lack of response in some patients with an RVEDV index below 90 ml/m^2^. Furthermore, the response to volume loading was rather unpredictable when RVEDV index ranged between these extremes. Based on those findings, no threshold value may be proposed to discriminate between responders and nonresponders before fluid application [18,21]. Nevertheless, most authors stated that thermodilution-derived estimates of RVEDV index appeared to be better indicators of cardiac preload [19,27,29] and can predict preload recruitable increases in SVI more accurately than can cardiac filling pressures [17,18].

In the present study ΔCEDV index was significantly correlated with ΔSVI_TD_, whereas there was a lack of correlations between changes in the remaining preload-indicating variables and ΔSVI_TD_, suggesting that increased cardiac preload is more reliably reflected by CEDV index than by CVP, PCWP, or LVEDA index. Some investigators questioned the clinical significance of correlation between RVEDV index and continuously assessed CI, but Durham [19] and Nelson [30] and their groups demonstrated that mathematical coupling does not account for the relationship between variables.

Several authors described RVEDV index as a marker of cardiac preload, indicated by the linear correlation between CI and RVEDV index [17-20]. Although a linear correlation between variables seems unlikely because measurements might have been performed at different operating points on the nonlinear curve describing the relationship between end-diastolic volume and stroke volume, the authors stated that RVEDV index could accurately predict preload recruitable increase in CI [17,18]. However, validation of a variable as an indicator of preload requires, in addition to demonstrations that the variable increases with fluid loading and that the increase is related to an increase in stroke volume, the demonstration that this variable does not change with an intervention that alters cardiac contractility (e.g. administration of inotropic agents). In most of studies preload-indicating variables were not tested in the presence of inotropic drugs, and therefore the hypothesis that RVEDV index is an accurate indicator of preload has not yet been proven. Accordingly, assuming that changes in myocardial contractility or afterload did not occur during the study period and measurements were performed in the steep part of the Frank–Starling curve, the significant relationship between CEDV index and CI in our study may merely indicate that an increased cardiac preload is reliably reflected by CEDV index.

It should be noted that the terms 'cardiac preload' and 'fluid responsiveness' are not exchangeable. The increase in SVI depends on ventricular function; a decrease in ventricular contractility decreases the slope of the relationship between end-diastolic volume and stroke volume [31] and moves the Frank–Starling curve to the right. Therefore, patients with a dilated left ventricle could still respond to fluid despite increased measures of static cardiac preload. Consequently, fluid responsiveness, defined as the response of SVI to volume challenge [32], cannot be accurately predicted simply by assessing cardiac preload.

For this reason, the more relevant question concerns the value of RVEDV index as an indicator of fluid responsiveness, but until now only limited and inconsistent information has been available regarding the value of this variable [20,22]. A variable is a predictor of fluid responsiveness if there is a relationship between the baseline value of that variable and changes in SVI after fluid loading. Reuse and coworkers [20] demonstrated a weak correlation (r^2 ^= 0.19; *P *< 0.01) between the response to fluid challenge and baseline RVEDV index in 41 critically ill patients. Wagner and Leatherman [22] found a comparable, modest correlation among variables (r^2 ^= 0.19; *P *< 0.05), but they stated that RVEDV index was not a reliable predictor of response to fluid.

In the present study, baseline values of CEDV index were not correlated with changes in SVI_TD _(Fig. 2a). Furthermore, using previously suggested criteria [15], neither a very high (>138 ml/m^2^) nor a very low (<90 ml/m^2^) CEDV index proved to be a reliable predictor of hemodynamic response to volume challenge. In accordance with Wagner et al [22], even one patient with markedly elevated CEDVI (159 ml/m2) was able to increase SVI in response to a fluid challenge in this study. This phenomenon may be accounted for by the fact that the left ventricular response to fluid loading may be predicted by the right ventricular volume only in a limited manner. The optimal CEDV index should be determined individually for each patient. Consequently, patients should not be resuscitated to an absolute CEDV index, but rather based upon their individual response of CEDV index and CCI to fluid administration.

A factor that could possibly affect the accuracy of CEDV index is the presence of a low RVEF [22], because CEDV index is calculated as the quotient of SVI and RVEF. The mean RVEF for the patients studied was 30.7 ± 9.1% at baseline, which is markedly lower than in the study conducted by Diebel and coworkers [18] (38 ± 9%). It is possible that CEDV index is a better predictor of response to volume in patients with higher RVEF. Another factor that should be taken into account was mentioned by Michard and coworkers [21,31]. The increase in ventricular end-diastolic volumes as a result of fluid challenge depends on the partitioning of fluid into different cardiovascular compartments organized in series. When ventricular capacitance is increased, volume loading will increase intravascular blood volume but not necessarily cardiac preload [31].

The results of the present investigation suggest that LVEDA index is a better predictor of fluid responsiveness than is CEDV index, and is even better than CVP or PCWP, as indicated by the weak correlation between baseline value of LVEDA index and the resulting increase in SVI_TD _following fluid loading (Fig. 2b). These findings are in accordance with those of other studies [7,13] and emphasize the importance of TEE in detecting acute changes in hemodynamics. However, the short-axis view provides only an area, not a volume, and the assumption that this area correlates with a volume is only valid when there are no regional contraction abnormalities [28,32]. The findings of recent studies demonstrate a limited relationship between hemodynamic and echocardiographic evaluation of left ventricular performance [33] and the minimal value of LVEDA index in discriminating responders from nonresponders [7]. The analysis presented in Fig. 2b shows the considerable influence of two data points corresponding to relative increases in SVI of about 77% and 94%. For the other patients, exhibiting relative increases in stroke volume of 10–40%, LVEDA index could not predict reliably the magnitude of this response. Furthermore, echocardiography requires an experienced investigator, is sometimes impossible to perform, and its availability as a device for continuous assessment of hemodynamics in the ICU is limited.

### Limitations

Monitoring of CEDV index can be unreliable in the presence of severe tricuspid valve insufficiency or during conditions of unsteady or rapid changing blood temperature. Furthermore, tachycardia at rates in excess of 150 beats/min will prevent accurate measurement of the patient's R–R interval.

For ethical reasons, assessment of the hemodynamic response of CEDV index was only be performed by a unidirectional preload change. Therefore, this parameter should be evaluated additionally under hemorrhage conditions in an animal experimental setting concerning its relative correctness.

In this setting of preoperative cardiac surgery, characterized by preoperative fasting, diuretic therapy, and the vasodilatory effect of sevoflurane, relative hypovolemia is common and could account for the fact that most of the patients responded to fluid. The small number of patients in the nonresponder group makes any conclusion regarding possible differences in any of the variables between responders and nonresponders difficult.

## Conclusion

Despite the limitations mentioned above, the results of the present study demonstrated that an increased cardiac preload is more reliably reflected by CEDV index than by CVP, PCWP or LVEDA index in this setting of preoperative cardiac surgery. However, CEDV index failed to be a variable of fluid responsiveness. The response of SVITD subsequent to fluid administration is better predicted by LVEDA index than by CEDV index.

## Key messages

• 	An increased cardiac preload is more reliably reflected by CEDV index than by CVP, PCWP or LVEDA index.

• 	But CEDV index did not reflect fluid responsiveness.

• 	The terms "cardiac preload" and "fluid responsiveness" are not exchangeable.

• 	Fluid responsiveness is better predicted by LVEDA index than by CEDV index.

## Abbreviations

CCO = continuous cardiac output; CEDV = continuous right ventricular end-diastolic volume; CI = cardiac index; CO = cardiac output; CVP = central venous pressure; HR = heart rate; ICU = intensive care unit; LVEDA = left ventricular end-diastolic area; PAC = pulmonary artery catheter; PCWP = pulmonary capillary wedge pressure; RVEF = right ventricular ejection fraction; RVEDV = right ventricular end-diastolic volume; SV = stroke volume; SVI_TD _= thermodilution-derived stroke volume index; TEE = transesophageal echocardiography.

## Competing interests

The author(s) declare that they have no competing interests.

## Authors' contributions

CW designed the study, processed the data, and wrote the manuscript. CF collected the clinical data. AK collected the clinical data and participated in the study design. SL collected the clinical data. CP designed the study and collected the clinical data. CK performed the statistical analysis and extensively revised the manuscript. All authors read and approved the final manuscript.

## Figures and Tables

**Table 1:** Demographic data and preoperative risk factors

Parameters	Value
Demographic data	
Age (years)	65.7 ± 6.1
Sex (male/female; % female)	17/4 (19.0%)
BMI (kg/m^2^)	29.1 ± 2.9
LVEF (%)	52.9 ± 13.7
LVEDP (mmHg)	12.9 ± 5.7
Preoperative risk factors (*n *[%])	
Diabetes	8 (38%)
Hypertension	17 (81%)
Smoking	6 (28%)
Hyperlipidemia	13 (62%)
Myocardial infarction	0 (0%)
Obesity	9 (43%)
History of stroke	2 (9.5%)
PVD	4 (19%)
PAH	0 (0%)
COPD	3 (14%)
Renal disease	4 (19%)

Data are expressed as mean ± standard deviation, or as frequency distributions (*n*) and simple percentages (%). BMI, body mass index; COPD = chronic obstructive pulmonary disease; LVEDP = left ventricular end-diastolic pressure; LVEF, left ventricular ejection fraction; PAH = pulmonary arterial hypertension; PVD = peripheral vascular disease.

**Table 2:** Hemodynamic variables at sample points T1 and T2

Variable	T1	T2	p-value
HR (beats/min)	62.2 ± 10.7	60.7 ± 8.4	NS
MAP (mmHg)	70.1 ± 10.9	82.2 ± 9.8	<0.01
CVP (mmHg)	10.9 ± 2.2	13.4 ± 2.3	<0.01
PCWP (mmHg)	11.6 ± 2.6	15.2 ± 2.4	<0.01
MPAP (mmHg)	20.5 ± 2.8	23.9 ± 4.1	<0.01
SVRI (dyne· s/cm^5^·m^2^)	2878 ± 698	2540 ± 394	<0.01
CI_TD _(l/min·m^2^)	1.70 ± 0.20	2.22 ± 0.23	<0.01
SVI_TD _(ml/m^2^)	28.3 ± 6.4	37.2 ± 6.1	<0.01
CCI (l/min·m^2^)	1.71 ± 0.26	2.20 ± 0.23	<0.01
CEDVI (ml/m^2^)	95.4 ± 21.0	122.1 ± 24.4	<0.01
RVEF (%)	30.7 ± 9.1	31.7 ± 8.4	NS
SvO_2 _(%)	73.5 ± 4.3	77.1 ± 3.8	<0.01
LVEDAI (cm^2^/m^2^)	11.1 ± 4.1	12.8 ± 4.6	<0.01
FAC (%)	38.2 ± 9.6	37.9 ± 8.4	NS

Times T1 and T2 are before volume replacement and 12 min after volume replacement, respectively. CCI, continuous cardiac index; CEDVI, continuous right ventricular end-diastolic volume index; CI, cardiac index; CVP, central venous pressure; FAC, fractional area change; HR, heart rate; LVEDAI, left ventricular end-diastolic area index; MAP, mean arterial pressure; MPAP, mean pulmonary arterial pressure; PCWP, pulmonary capillary wedge pressure; SVI, stroke volume index; SVRI, systemic vascular resistance index; RVEF, right ventricular ejection fraction; SvO_2_, mixed venous oxygen saturation.
